# Death of Dr. V. H. Taliaferro

**Published:** 1887-10

**Authors:** 


### DEATH OF DR. V. H. TALIAFERRO.

In the death of Professor V. H. TALIAFERRO, which occurred since our last issue, at Tate Springs, the Medical College of Atlanta and the profession in the city have sustained a great loss.

Entering the Confederate States army as a private soldier, he served throughout the war between the States with such fidelity and distinction that by successive promotions he attained the command of his regiment, and at its close was an acting brigadier-general.

After the close of the war, he devoted himself to the practice of medicine with patience, diligence and success, and in a few years was elected professor of obstetrics in the Medical College of Atlanta. As a teacher, he was able, clear and accurate. The duties of his position naturally guided his studies and practice in the direction of a specialty, in which he attained eminence and fame. He sought not only to teach what was already known and accepted as true, but to enlarge the boundaries of science, and by his inventive genius to devise new expedients for the cure of disease or the mitigation of suffering.

It is hard to estimate the loss to humanity in the early death of such a man in the midst of his usefulness and in the full maturity of his intellect.

